# Two roads to lignin: uncovering the role of C4H in rice lignification

**DOI:** 10.1093/plphys/kiaf233

**Published:** 2025-06-05

**Authors:** Guannan Wang

**Affiliations:** Plant Physiology, American Society of Plant Biologists; Department of Biology, Stanford University, Stanford, CA 94305, USA; Howard Hughes Medical Institute, Stanford University, Stanford, CA 94305, USA

Lignin constitutes approximately 30% of the organic carbon on Earth and is recognized as the second most abundant terrestrial biopolymer after cellulose (Boerjan et al. 2003). Lignin is deposited predominantly in the secondary cell walls of vascular plants and provides mechanical strength, structural rigidity, and hydrophobicity to plant vascular tissues (Liu and Eudes 2022). The emergence of lignin and its incorporation into cell walls has long been considered as one of the key innovations in land plants that facilitated their rapid diversification and dominance of the terrestrial ecosystem (Weng and Chapple 2010).

Lignin is synthesized through the oxidative polymerization of 3 primary *p*-hydroxycinnamyl alcohols (monolignols): *p*-coumaryl, coniferyl, and sinapyl alcohols, resulting in *p*-hydroxyphenyl (H), guaiacyl (G), and syringyl (S) units, respectively (Tobimatsu and Schuetz 2019). These monolignols are derived from the general phenylpropanoid pathway, which typically starts with the aromatic amino acid phenylalanine produced by the shikimate pathway (Vanholme et al. 2010). The initial committed steps in this pathway involve the deamination of phenylalanine to trans*-*cinnamic acid by phenylalanine ammonia-lyases, followed by hydroxylation to *p*-coumaric acid catalyzed by the cytochrome P450 enzyme cinnamate 4-hydroxylase (C4H) (Fig. 1) (Vogt 2010). However, grasses in the Poaceae family possess an alternative route to phenylpropanoid precursors, in which bifunctional phenylalanine/tyrosine ammonia-lyases (PTALs) directly deaminate tyrosine to produce *p*-coumaric acid, thereby bypassing the canonical phenylalanine ammonia-lyase (PAL)-C4H pathway (Fig. 1) (Barros and Dixon 2020). How the PAL-C4H pathway contributes to lignin biosynthesis in grasses, where a parallel tyrosine-derived route is also active, remains unclear. In this issue of *Plant Physiology*, Supatmi et al. (2025) used rice as a model grass species to investigate the role of C4H in cell wall lignification.

**Figure 1.**
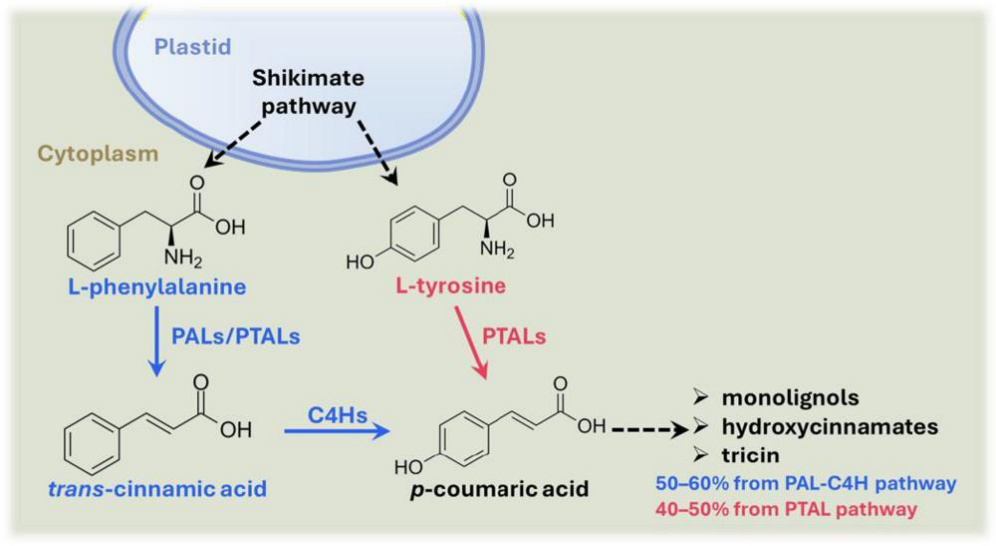
Proposed model for the early steps of lignin synthesis in rice. The canonical PAL-C4H pathway and the alternative PTAL pathway are highlighted in blue and red, respectively.

An early duplication event of C4H occurred prior to the split between gymnosperms and angiosperms, giving rise to class I and class II C4H in most seed plants (Renault et al. 2017). A second gene duplication event of C4H occurred in monocots (Hansen et al. 2021). As a consequence, multiple copies of C4H genes from both classes have been identified in the rice genome: *CYP73A35p*, *CYP73A38* (*C4H1*) from class I and *CYP73A39* (*C4H2a*), *CYP73A40* (*C4H2b*) from class II. Among those, *CYP73A35p* is predicted to encode a nonfunctional C4H due to the lack of functional domains and undetectable gene expression. The class I *OsC4H1* is expressed broadly across major vegetative organs, whereas class II *C4H*s are predominantly expressed in roots. To investigate the functional roles of these *C4H*s, the authors generated a single knockout line for *OsC4H1* (*OsC4H1-KO*), a double knockout line for both *OsC4H2a* and *OsC4H2b* (*OsC4H2a/2b-DKO*), and a triple knockout line disrupting all 3 genes (*OsC4H1/2a/2b-TKO*).

All the mutant lines exhibited significant reductions in shoot height, shoot biomass, and root biomass during the seedling stage, as well as in culm (essentially the rice stem) length when reaching maturation. The plant height was reduced in all mutants but only significantly in the triple knockdown mutants, and seed fertility was dramatically decreased only in mutants where *OsC4H1* was disrupted. No anatomical abnormalities were detected in the culms of *OsC4H*-deficient mutants, except for the reduced lignin content and smaller lignified vascular bundles.

The authors further found that not only was total lignin content reduced, but levels of major lignin monomers, cell wall–bound *p*-coumaric acid and ferulate, and lignin-bound tricin were also significantly decreased in the culms from *OsC4H1*-deficient mutants. In contrast, decreased lignin levels in root were only detected in the triple knockout mutants where all *C4H*s were mutated. The distinct effects on lignin content in culms versus roots across different mutant backgrounds underscore the organ-specific functional specialization of *OsC4H*s in rice. The proportion of H-, G-, and S-type lignin monomers, as well as the abundance of most cell wall monosaccharides, largely remained unaffected in all mutants. These findings demonstrate that the 3 *OsC4H*s collectively contribute to the synthesis of lignin, hydroxycinnamates, and tricin in rice with partially redundant and organ-specific roles.

Intriguingly, compared to *C4H*-deficient eudicots, disruption of *C4H*s in rice resulted in generally much milder effects on plant growth and did not completely abolish the biosynthesis of lignin precursors. As mentioned above, grasses could bypass the canonical PAL-C4H pathway by using PTALs to synthesize phenylpropanoid precursor *p*-coumaric acid directly from tyrosine (Fig. 1) (Barros and Dixon 2020). Therefore, the authors investigated the relative contributions of dual PAL-C4H and PTAL pathways to lignin biosynthesis in their rice mutants. No significant differences in PAL and TAL activities were observed between the wild-type rice and the triple knockout mutants. Treatment with piperonylic acid, an inhibitor for C4H, had no effect on plant growth or lignin levels in the triple knockout mutants. However, treatment with L-2-aminooxy-3-phenylpropionic acid, which inhibits both PAL and TAL activities, led to a further reduction in plant growth and lignin content in the triple knockout mutants. When supplemented with radiolabeled phenylalanine and tyrosine, wild-type rice exhibited consistently higher incorporation of radiolabeled phenylalanine than radiolabeled tyrosine into major lignin and hydroxycinnamate units, indicating a dominant contribution of PAL-C4H pathway to lignin biosynthesis in rice (Fig. 1). The triple knockout mutants, in which the PAL-C4H pathway is entirely blocked, failed to incorporate any radiolabeled phenylalanine but maintained the incorporation of tyrosine at levels comparable to that in the wild-type rice. These results together suggest that the PTAL pathway can substantially compensate for the loss of the PAL-C4H pathway in rice.

In summary, the work by Supatmi et al. (2025) revealed that while *C4H*s are essential for cell wall lignification in rice, their function is largely dispensable due to the presence of the alternative PTAL pathway, which can compensate for the complete blockage of the canonical PAL-C4H pathway. The 2 classes of *C4H*s exhibit distinct expression patterns and differentially influence plant growth during vegetative and reproductive stages. Therefore, further investigation will be needed to clarify the functional divergence between these *C4H*s and their contributions to cell wall lignification across diverse cell types and environmental contexts. Given that grass lignin exhibits remarkable structural diversity compared to that of eudicots (Barros and Dixon 2020), it will be particularly interesting to investigate the respective role of the PAL-C4H and PTAL pathways in generating this diversity, as well as to explore its evolutionary significance in grasses. Addressing these questions will not only provide deeper insights into how land plants adapted to the terrestrial environment but will also greatly facilitate precise engineering of desirable lignin traits in grasses for sustainable production of biofuels and biomass-based materials.

## Data Availability

No new data were generated or analysed in support of this research.

## References

[kiaf233-B1] Barros J, Dixon RA. Plant phenylalanine/tyrosine ammonia-lyases. Trends Plant Sci. 2020:25(1):66–79. 10.1016/j.tplants.2019.09.011 31679994

[kiaf233-B2] Boerjan W, Ralph J, Baucher M. Lignin biosynthesis. Annu Rev Plant Biol. 2003:54(1):519–546. 10.1146/annurev.arplant.54.031902.134938 14503002

[kiaf233-B3] Hansen CC, Nelson DR, Møller BL, Werck-Reichhart D. Plant cytochrome P450 plasticity and evolution. Mol Plant. 2021:14(8):1244–1265. 10.1016/j.molp.2021.06.028 34216829

[kiaf233-B4] Liu C-J, Eudes A. Lignin synthesis and bioengineering approaches toward lignin modification. Adv Bot Res. 2022:104:41–96. 10.1016/bs.abr.2022.02.002

[kiaf233-B5] Renault H, De Marothy M, Jonasson G, Lara P, Nelson DR, Nilsson I, André F, von Heijne G, Werck-Reichhart D. Gene duplication leads to altered membrane topology of a cytochrome P450 enzyme in seed plants. Mol Biol Evol. 2017:34(8):2041–2056. 10.1093/molbev/msx160 28505373 PMC5850782

[kiaf233-B6] Supatmi S, Lam LPY, Yamamoto S, Afifi OA, Ji P, Osakabe Y, Osakabe K, Umezawa T, Tobimatsu Y. Essential yet dispensable: the role of CINNAMATE 4-HYDROXYLASE in rice cell wall lignification. Plant Physiol. 2025:198(1):kiaf164. 10.1093/plphys/kiaf164 40272427

[kiaf233-B7] Tobimatsu Y, Schuetz M. Lignin polymerization: how do plants manage the chemistry so well? Curr Opin Biotechnol. 2019:56:75–81. 10.1016/j.copbio.2018.10.001 30359808

[kiaf233-B8] Vanholme R, Demedts B, Morreel K, Ralph J, Boerjan W. Lignin biosynthesis and structure. Plant Physiol. 2010:153(3):895–905. 10.1104/pp.110.155119 20472751 PMC2899938

[kiaf233-B9] Vogt T . Phenylpropanoid biosynthesis. Mol Plant. 2010:3(1):2–20. 10.1093/mp/ssp106 20035037

[kiaf233-B10] Weng J-K, Chapple C. The origin and evolution of lignin biosynthesis. New Phytol. 2010:187(2):273–285. 10.1111/j.1469-8137.2010.03327.x 20642725

